# Common surgical procedures in pilonidal sinus disease: A meta-analysis, merged data analysis, and comprehensive study on recurrence

**DOI:** 10.1038/s41598-018-20143-4

**Published:** 2018-02-15

**Authors:** V. K. Stauffer, M. M. Luedi, P. Kauf, M. Schmid, M. Diekmann, K. Wieferich, B. Schnüriger, D. Doll

**Affiliations:** 1 Lindenhofspital, Lindenhofgruppe, 3010 Bern, (VS) Switzerland; 2 Department of Anaesthesiology, Bern University Hospital Inselspital, University of Bern, 3010 Bern, (MML) Switzerland; 3 Biomedical Statistics PROGNOSIX AG, 8001 Zurich, (PK, MS) Switzerland; 4 Department of Procto-Surgery, St. Marien-Krankenhaus Vechta, Teaching Hospital of the Hannover University, 49377 Vechta, (KW, DD) Germany; 5 Department of Visceral Surgery and Medicine, Bern University Hospital Inselspital, University of Bern, 3010 Bern, (BS) Switzerland

## Abstract

We systematically searched available databases. We reviewed 6,143 studies published from 1833 to 2017. Reports in English, French, German, Italian, and Spanish were considered, as were publications in other languages if definitive treatment and recurrence at specific follow-up times were described in an English abstract. We assessed data in the manner of a meta-analysis of RCTs; further we assessed non-RCTs in the manner of a merged data analysis. In the RCT analysis including 11,730 patients, Limberg & Dufourmentel operations were associated with low recurrence of 0.6% (95%CI 0.3–0.9%) 12 months and 1.8% (95%CI 1.1–2.4%) respectively 24 months postoperatively. Analysing 89,583 patients from RCTs and non-RCTs, the Karydakis & Bascom approaches were associated with recurrence of only 0.2% (95%CI 0.1–0.3%) 12 months and 0.6% (95%CI 0.5–0.8%) 24 months postoperatively. Primary midline closure exhibited long-term recurrence up to 67.9% (95%CI 53.3–82.4%) 240 months post-surgery. For most procedures, only a few RCTs without long term follow up data exist, but substitute data from numerous non-RCTs are available. Recurrence in PSD is highly dependent on surgical procedure and by follow-up time; both must be considered when drawing conclusions regarding the efficacy of a procedure.

## Introduction

For unknown reasons, the incidence of pilonidal sinus disease (PSD) has risen continuously during the past 50 years, particularly in European and North American young men^1,2^. In a German military cohort for example, the number of affected patients increased from 29/100,000 in 2000 to 48/100,000 in 2012, and the total number of PSD-related in-patient surgeries exceeded the number of inguinal hernia-related interventions in 20 to 40-year-old patients^3^. Recurrent disease may probably affect patients’ long-term satisfaction following PSD surgery^4^. Recurrence between 0 percent^5^ and 100 percent^6^ has been reported for PSD, and wide recurrence range can be seen even within the different surgical approach techniques as open treatment, primary midline closure or flap techniques and others. Some evidence suggests that recurrence is associated with surgical procedure and correlated with length of follow-up as well^4,7^. However, the data are conflicting, and applied follow-up times often appear to have been randomly chosen, which brings into question the validity of reported recurrence associated with different surgical procedures. The purpose of this meta-analysis and merged data analysis was therefore to obtain a comprehensive assessment of recurrence and to ascertain determinants of recurrence of PSD with respect to specific surgical procedures and follow-up time. We considered both randomised controlled trials (RCTs) and non-RCTs.

We thus assembled a database with sources from the first description of PSD in 1833 on, that included reported recurrence, year of publication, timeframes of follow-up, type of study, and patient- and procedure-specific factors. We grouped therapeutic procedures for cumulative statistical analyses (Table 1). Using this dataset, we assessed the efficacy of common surgical procedures employed in treating PSD as a function of recurrence. We found that the recurrence in PSD varied depending on the surgical procedure and on the length of follow-up. While naturally, an increase of recurrence could be observed with longer follow-up, the rate of this increase was varying among the different procedures. This indicates that a thorough evaluation of a procedure in view of recurrence has to include the specific relation of recurrence to follow-up time and cannot just be based on comparisons at one single follow-up time. The strength of our conclusions is substantially buttressed by the extensive analysis of a large database pertaining to particular therapeutic procedures.

**Table 1:** Grouping of therapeutic strategies for analysis of recurrence rates in pilonidal sinus disease.

*Primary open*	Excision, “exhairese”, vacuum assisted closure (VAC), sinusectomy/excision, atypical excision and any other primary open approaches including supplemental measures such as laser, phenol, cryotherapy, local or systemic antibiotics, and platelet rich plasma in wound
*Primary median closure*	Any primary midline closure approach including supplemental measures such as laser, phenol, cryotherapy, local antibiotics, drainage, wound closure over antibiotics (“all put in closed wound”), systemic antibiotics, platelet rich plasma in wound but not using advancement or rotation flap techniques
*Primary asym. closure*	S-shape closure, D-shape closure, D-flap, oblique crossing, Casten and modified Casten approach
*Karydakis*/Bascom*	Bascom cleft lift*, and modified Bascom cleft lift* approach, Karydakis and modified Karydakis approach, cleft lift procedure, including supplemental measures such as laser, phenol, cryotherapy, local antibiotics, drainage, wound closure over antibiotics (“all put in closed wound”), systemic antibiotics, platelet rich plasma in wound
*Limberg*/*Dufourmentel*	Limberg and Dufourmentel approach as well as their modifications, rhomboid flap, teardrop flap and z-plasty including supplemental measures such as local or systemic antibiotics
*Flaps*	Classical advancement flap, gluteus flap, VY-advancement flap, lateral advancement flap, local fasciocutaneous, infragluteal, and bilateral gluteus muscle advancement flap, “lembo di lalor”, pope musculofascial advancement flap, “Kopp gluteo-fascial plasty”, Rotation flap, Schrudde-Olivari and other flaps including combinations and supplemental measures such as local or systemic antibiotics
*Marsupialisation*	Marsupialisation as described by Obeid, McFee, Mutschmann, DePrizio, Colp and Buie
*Limited excision*	Lay open, curettage, drainage, sinotomy, sinotomy and cauterisation, “cystostomie”, minor excision, curettage, deroofing and curettage, cauterisation, and flush as described by Dorton
Pit picking*	Bascom pit picking with a lateral incision *, Trephines, pit picking, pit excision, pit excision and phenol, brushing, Farrell drills,Lord-Millar, primary open approach with subcutaneous excision of collateral tracts, tract coagulation
*Partial closure*	Partial closure techniques including supplemental measures such as local or systemic antibiotics
*Incision and drainage*	Incision, incision and curettage, and aspiration including supplemental measures such as, local or systemic antibiotics
*Phenol treatment*	Classic phenol treatment and supplemental measures such as laser, cryotherapy, and local or systemic antibiotics
*Laser treatment*	Primary laser techniques
*Other treatments additionally included in the overall analysis*	Plug and Seton technique, as well as endoscopic approaches, cryotherapy, histoacryl glue injection, aspiration and antibiotics, and conservative approaches such as Ayurveda therapy

^*^Bascom described and used two different procedures: “Cleft closure/cleft lift” (merged with Karydakis group) and “Pit picking” (merged with Pit picking group).

## Results

**Figure 1:**
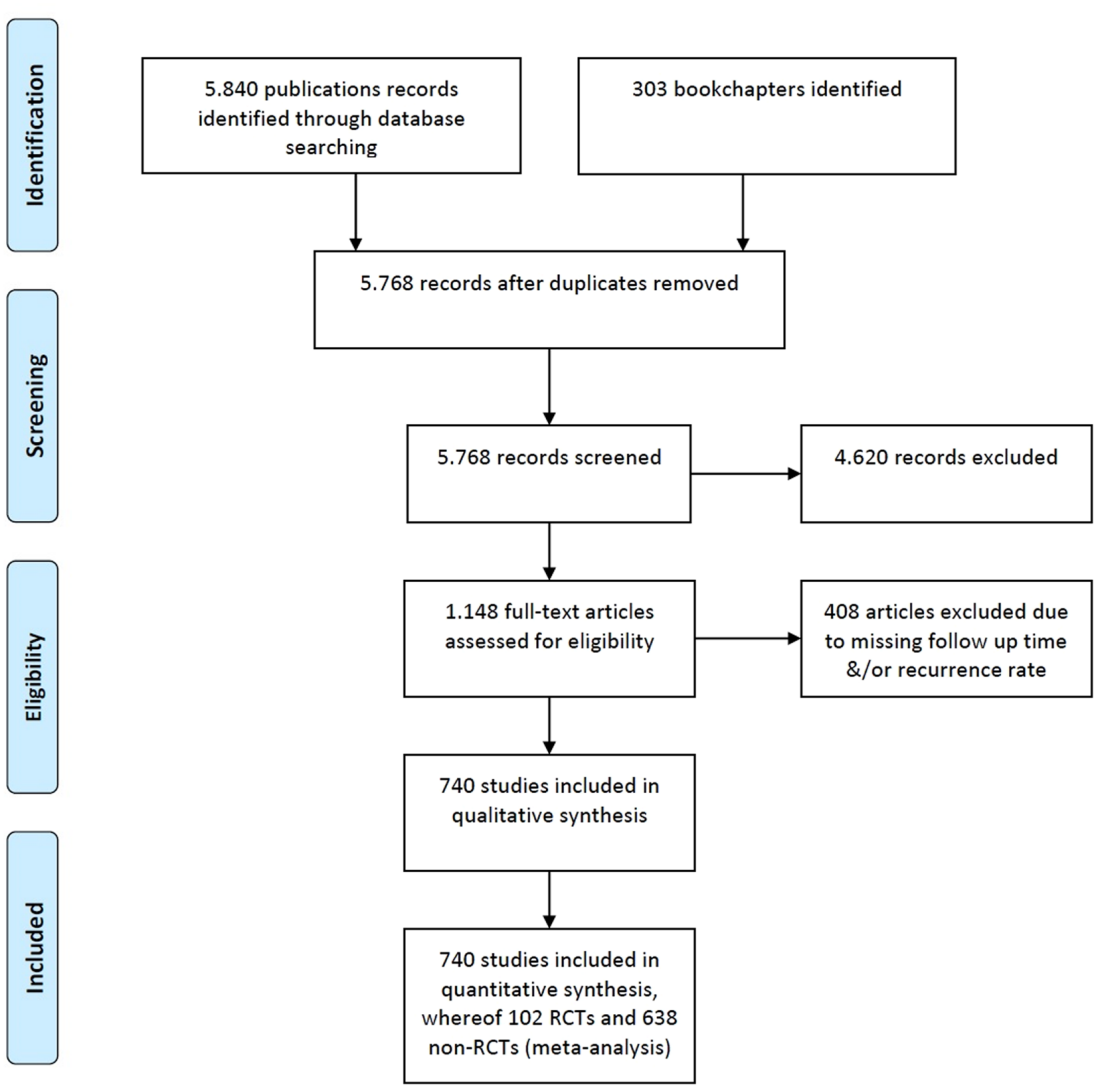
Flow diagram based on *Preferred reporting items for systematic reviews and meta-analysis (PRISMA)*^8^ illustrating the systematic search for evidence regarding recurrence and long term follow-up data associated with common surgical procedures in PSD.

Our search criteria retrieved 5,840 studies and 303 book chapters across all databases. After excluding duplicates, 5,768 studies were screened. Reports on PSD in other than the classical presacral intergluteal location, studies in embryonic development, in carcinomas, etc. were excluded. Following exclusion, 1,148 articles on PSD at classical anatomical location with specific surgical treatment remained for analysis. Of these, 408 lacked detailed data on recurrence or follow-up time or both. Finally, 740 studies published from 1833 to 2017 were analysed. A flow chart describing the selection of literature sources, based on the *Preferred reporting items for systematic reviews and meta-analysis (PRISMA)*^8^, is illustrated in Fig. 1.

Results reported in the final set of publications were stratified according to the specific surgical technique employed to avoid bias across studies. This approach led to 14 groups for analysis. Additionally, we included an overall analysis. For each of the specific therapeutic approaches, the data included the number of patients, the reported follow-up time, and the recurrence.

### Heterogeneity analysis

Considering prospective/randomized control trials only, the heterogeneity analysis showed I^2^ < 5%, p > 0.2 (Cochrane’s Q-test) except for the Bascom/Karydakis (0–12 months, p < 0.001, I^2^ = 80.36%, df = 4), marsupialisation (0–12 months; p < 0.001, I^2^ = 97.84%, df = 3), and other flap techniques (0–12 months, p = 0.062, I^2^ = 64%, df = 2). Considering all studies, the heterogeneity analysis showed I^2^ < 5%, p > 0.2 (Cochrane’s Q-test) except for the primary asymmetric closure (0–12 months, p = 0.023, I^2^ = 61.54%, df = 5), marsupialisation (0–12 months, p < 0.001, I^2^ = 64.43%, df = 18), and pit picking (0–12 months, p < 0.001, I^2^ = 98.31%, df = 6).

The above described analysis ascertains that there is no statistical evidence of heterogeneity in our study group, except for primary asymmetric closure, marsupialisation and pit picking.

### Follow-up time and recurrence over all surgical therapies

Data on recurrence and follow-up times in all surgical PSD treatments together pertaining to 11,700 patients were extracted from 102 RCTs^2,5,9–110^. A recurrence of 1.5% (95% CI 1.3–1.8%) was observed in patients at 12 months, 4.3% (95% CI 3.8–4.8%) at 24 months, and 20.3% (95% CI 17.8–22.9%) at 60 months.

Further, data on recurrence and follow-up times in all surgical PSD treatments together pertaining to primary open PSD treatment pertaining to a total of 89,583 patients were extracted from 638 additional non-RCTs^4,62,111–746^. Among these patients, a recurrence of 2.0% (95% CI 1.9–2.1%) was observed in patients at 12 months, 4.4% (95% CI 4.3–4.6%) at 24 months, 10.8% (95% CI 10.5–11.3%) at 60 months, 16.9% (95% CI 16.3–17.5%) at 120 months, and 60.4% (95% CI 47.1–37.8%) at 240 months (Fig. 4).

**Figure 2:**
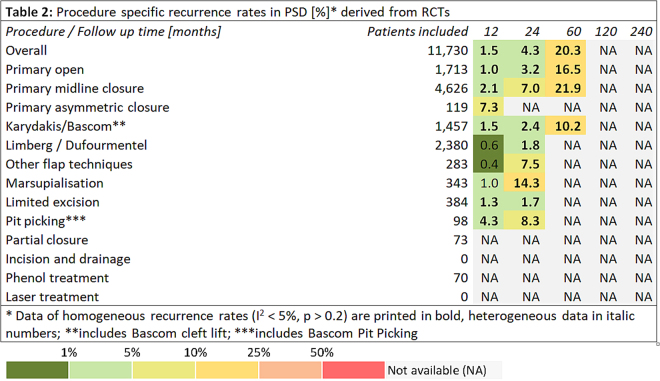
Procedure specific recurrence rates in PSD [%]* derived from RCTs. *Data of homogeneous recurrence rates (I^2^ < 5%, p > 0.2) are printed in bold, heterogeneous data in italic numbers; **includes Bascom cleft lift; ^***^includes Bascom Pit Picking.

**Figure 3:**
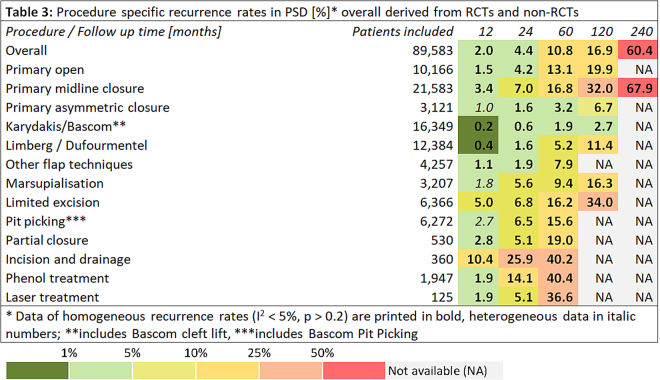
Procedure specific recurrence rates in PSD [%]* overall derived from RCTs and non-RCTs. *Data of homogeneous recurrence rates (I^2^ < 5%, p > 0.2) are printed in bold, heterogeneous data in italic numbers; **includes Bascom cleft lift, ***includes Bascom Pit Picking.

**Figure 4:**
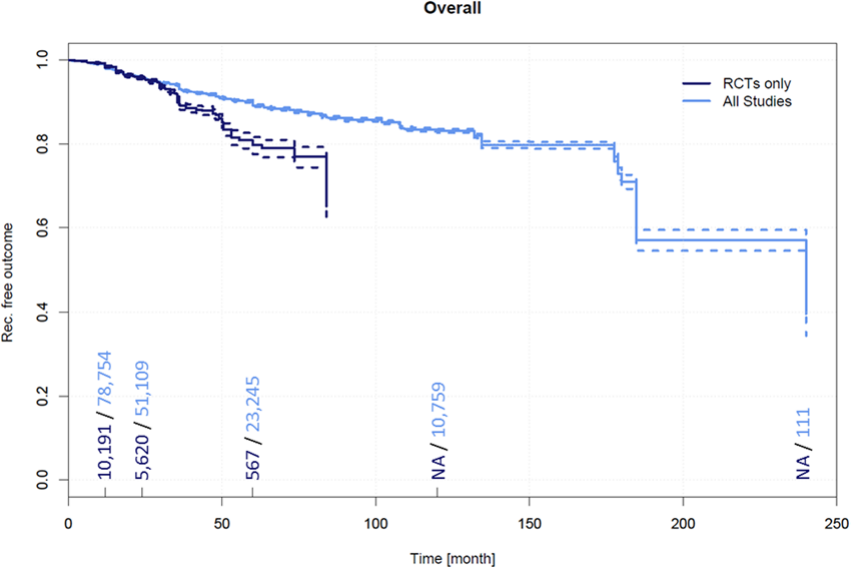
Recurrence free outcome as a function of follow-up time irrespective of specific therapeutic procedure. Data presented are for RCTs only and for all available studies. Numbers of patients included in the analysis are indicated at 12, 24, 60, and 120 months. Dashed lines indicate 95% confidence intervals.

To enable an entire picture, this overall analysis included data on recurrence and follow-up times pertaining to 184 patients treated for PSD by other methods (Table 1) extracted from 3 RCTs. Among these patients, a recurrence of 3.8% (95% CI 0.9–6.7%) was observed in patients at 12 months and 7.8% (95% CI 0.4–15.1%) at 24 months and follow-up times pertaining to 2,916 patients treated for PSD by other methods extracted from 40 additional non-RCTs. Among these patients, a recurrence of 2.9% (95% CI 2.2–3.7%) was observed in patients at 12 months, 6.7% (95% CI 5.4–8.0%) at 24 months, and 26.0% (95% CI 22.6–29.4%) at 60 months.

To provide a rational basis for selecting treatment approaches, we assessed possible associations between recurrence of PSD and specific therapeutic procedures in the manner of a classical meta-analysis of RCTs, and found that recurrence in common surgical procedures for PSD were dependent on follow-up time (Figure 2); additional data from non-RCTs were included and processed in the manner of a merged data analysis (Figure 3).

### Recurrence in primary open PSD treatment

Data on recurrence and follow-up times in primary open PSD treatment pertaining to 1,713 patients were extracted from 32 RCTs^9–38,747,748^. Among these patients, a recurrence of 1.0% (95% CI 0.5–1.6%) was observed in patients at 12 months, 3.2% (95% CI 2.2–4.2%) at 24 months, and 16.5% (95% CI 11.9–21.2%) at 60 months.

**Figure 5:**
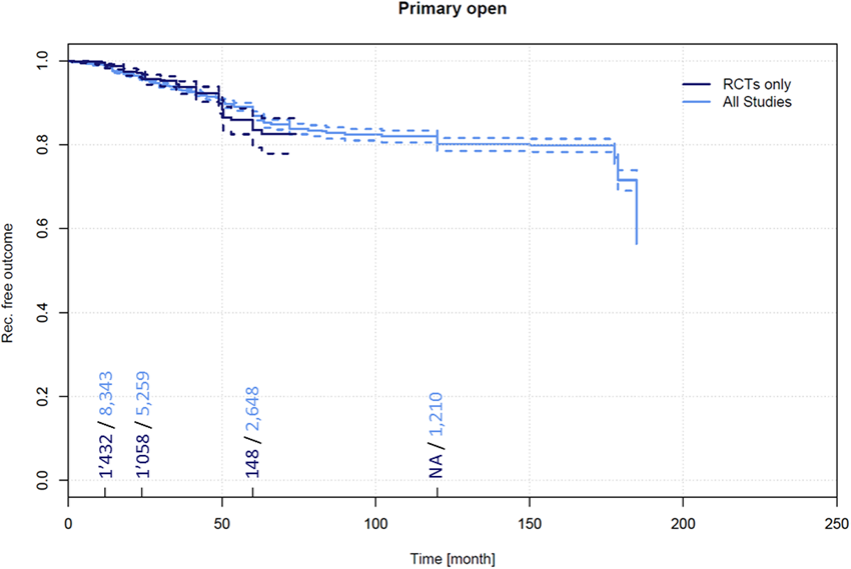
Recurrence free outcome as a function of follow-up time of patients receiving primary open treatment. Data presented are for RCTs only and for all available studies. Numbers of patients included in the analysis are indicated at 12, 24, 60, and 120 months. Dashed lines indicate 95% confidence intervals.

Further, data on recurrence and follow-up times in primary open PSD treatment pertaining to 10,166 patients were extracted from 128 additional non-RCTs^4,39,40,111–234^. Among these patients, a recurrence of 1.5% (95% CI 1.2–1.7%) was observed in patients at 12 months, 4.2% (95% CI 3.7–4.7%) at 24 months, 13.1% (95% CI 11.9–14.4%) at 60 months, and 19.9% (95% CI 17.9–21.9%) at 120 months (Fig. 5).

### Recurrence in primary midline closures

Data on recurrence and follow-up times in primary midline closures (not using advancement or rotation flap techniques) pertaining to 4,626 PSD patients which were extracted from 51 RCTs^5,9,10,19,20,22,25–29,31,32,35,37,41–73,235,236,748^. Among these patients, a recurrence of 2.1% (95% CI 1.7–2.6%) was observed in patients at 12 months, 7.0% (95% CI 6.0–8.0%) at 24 months, and 21.9% (95% CI 18.5–25.3%) at 60 months.

**Figure 6:**
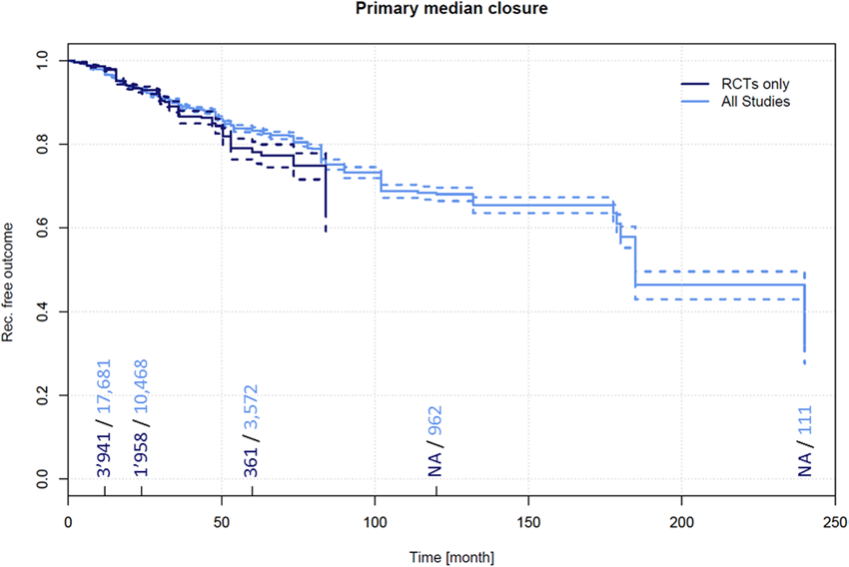
Recurrence free outcome as a function of follow-up time of patients treated with primary midline closure (not using advancement or rotation flap techniques). Data presented are for RCTs only and for all available studies. Numbers of patients included in the analysis are indicated at 12, 24, 60, and 120 months. Dashed lines indicate 95% confidence intervals.

Data on recurrence and follow-up times in primary open PSD treatment pertaining to 21,583 patients were extracted from 205 additional non-RCTs^4,60,74,75,111,112,114,115,117,118,121–126,128–134,136,137,141,143,149,153–156,160,161,163,167–171,174,175,177,181,182,188–192,194,196,197,199–201,203,206,208,215,216,218,220,221,223,224,230,233,237–373^. Among these patients, a recurrence of 3.4% (95% CI 3.1–3.6%) was observed in patients at 12 months, 7.0% (95% CI 6.5–7.4%) at 24 months, 16.8% (95% CI 15.8–17.8%) at 60 months, 32.0% (95% CI 29.6–34.4%) at 120 months, and 67.9% (95% CI 53.3–82.4%) at 240 months (Fig. 6).

### Recurrence in primary asymmetric closure

Data on recurrence and follow-up times in primary asymmetric closure PSD treatment pertaining to 119 patients were extracted from 2 RCTs^34,374^. Among these patients, a recurrence of 7.3% (95% CI 0.0–19.9%) was observed in patients at 12 months.

**Figure 7:**
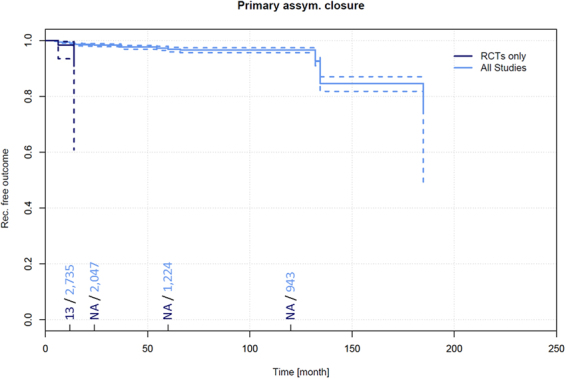
Recurrence free outcome as a function of follow-up time of patients treated with primary asymmetric closure. Data presented are for RCTs only and for all available studies. Numbers of patients included in the analysis are indicated at 12, 24, 60, and 120 months. Dashed lines indicate 95% confidence intervals.

Further, data on recurrence and follow-up times pertaining to 3,121 patients receiving primary open PSD treatment were extracted from 28 additional non-RCTs^4,76,130,133,139,157,221,228,320,348,357,375–391^. Among these patients, a recurrence of 1.0% (95% CI 0.6–1.4%) was observed in patients at 12 months, 1.6% (95% CI 1.1–2.1%) at 24 months, 3.2% (95% CI 2.3–4.0%) at 60 months, and 6.7% (95% CI 5.2–8.2%) at 120 months (Fig. 7).

### Recurrence in Karydakis and Bascom cleft lift techniques

Data on recurrence and follow-up times pertaining to 1,457 patients treated for PSD by a Karydakis or Bascom cleft lift technique were extracted from 21 RCTs^24,33,59,62,63,77–90,392,393^. Among these patients, a recurrence of 1.5% (95% CI 0.8–2.2%) was observed in patients at 12 months, 2.4% (95% CI 1.4–3.3%) at 24 months, and 10.2% (95% CI 5.4–15.0%) at 60 months.

**Figure 8:**
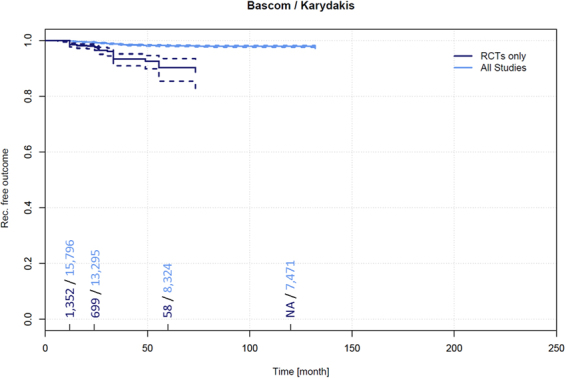
Recurrence free outcome as a function of follow-up time of patients treated with Bascom and Karydakis techniques. Data presented are for RCTs only and for all available studies. Numbers of patients included in the analysis are indicated at 12, 24, 60, and 120 months. Dashed lines indicate 95% confidence intervals.

Data on recurrence and follow-up times pertaining to 16,349 patients treated for PSD by a Karydakis and Bascom cleft lift technique were extracted from 66 additional non-RCTs^91,92,136,146,161,163,184,198,206,254,263,313,335,346,348,361,379,391,394–441^. Among these patients, a recurrence of 0.2% (95% CI 0.1–0.3%) was observed in patients at 12 months, 0.6% (95% CI 0.5–0.8%) at 24 months, 1.9% (95% CI 1.6–2.2%) at 60 months, and 2.7% (95% CI 2.4–3.1%) at 120 months (Fig. 8). Along with the Limberg/Dufourmentel approaches and other flap techniques, the Karydakis and Bascom cleft lift procedures resulted in the lowest recurrence at any follow-up time in our analysis (Tables 2 and 3).

### Recurrence in Limberg and Dufourmentel flaps

Data on recurrence and follow-up times pertaining to 2,380 patients treated for PSD by Limberg and Dufourmentel flap techniques were extracted from 36 RCTs^5,14,21,23,43,44,46,49,53,54,56,61,64,65,73,77,78,80,82,83,90,93–102,236,392,393,442,443^. Among these patients, a recurrence of 0.6% (95% CI 0.3–0.9%) was observed in patients at 12 months and 1.8% (95% CI 1.1–2.4%) at 24 months.

**Figure 9:**
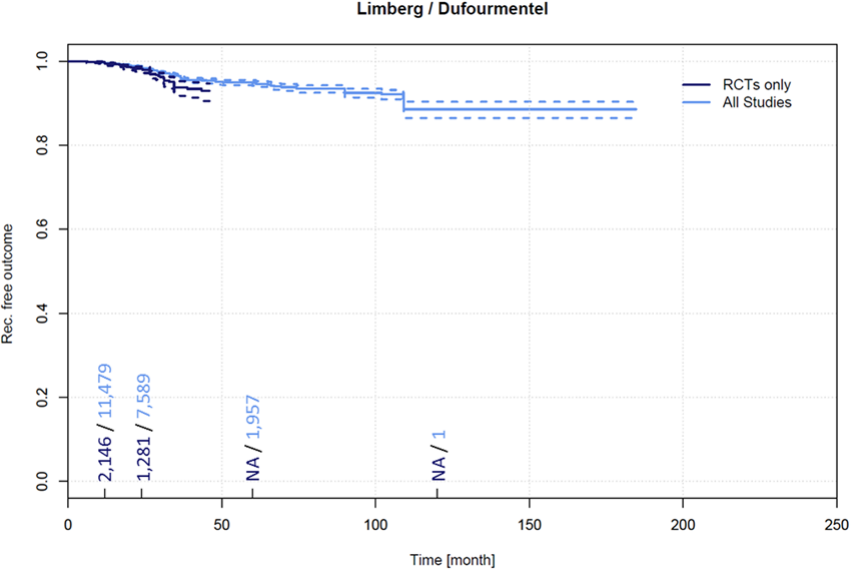
Recurrence free outcome as a function of follow-up time of patients treated with Limberg and Dufourmentel flap technique. Data presented are for RCTs only and for all available studies. Numbers of patients included in the analysis are indicated at 12, 24, 60, and 120 months. Dashed lines indicate 95% confidence intervals.

Data on recurrence and follow-up times pertaining to 12,384 patients treated for PSD by Limberg and Dufourmentel flaps were extracted from 139 additional non-RCTs^4,60,103,104,115,126–128,133,156,157,196,199,201,208,221,224,242,261,262,267,289,302,303,313,318,321,322,334–336,348,372,399,407,410,415,417,426,433,438,439,444–541^. Among these patients, a recurrence of 0.4% (95% CI 0.3–0.5%) was observed in patients at 12 months, 1.6% (95% CI 1.3–1.9%) at 24 months, 5.2% (95% CI 4.5–5.8%) at 60 months, and 11.4% (95% CI 9.2–13.7%) at 120 months (Fig. 9).

### Recurrence in other flap techniques

Data on recurrence and follow-up times pertaining to 283 patients treated for PSD by other flap techniques were extracted from 6 RCTs^45,55,98,105,542,543^. Among these patients, a recurrence of 0.4% (95% CI 0.0–1.1%) was observed in patients at 12 months and 7.5% (95% CI 2.4–12.5%) at 24 months.

**Figure 10:**
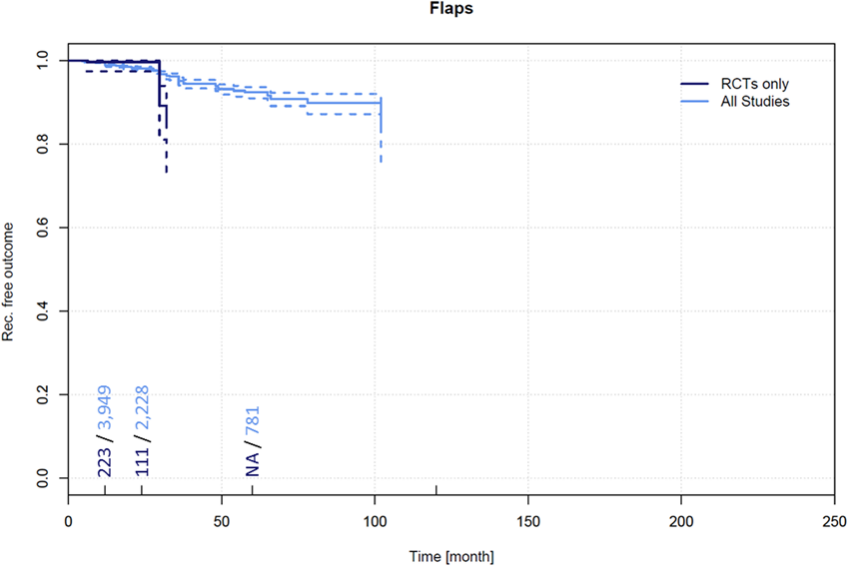
Recurrence free outcome as a function of follow-up time of patients treated with other flap techniques. Data presented are for RCTs only and for all available studies. Numbers of patients included in the analysis are indicated at 12, 24, 60, and 120 months. Dashed lines indicate 95% confidence intervals.

Data on recurrence and follow-up times pertaining to 4,258 patients treated for PSD by other flap techniques were extracted from 89 additional non-RCTs^106,107,111,126,137,160,167,182,192,208,221,224,233,245,259,283,312,321,339,354,368,410,433,449,492,511,512,525,532,538,544–602^. Among these patients, a recurrence of 1.1% (95% CI 0.8–1.4%) was observed in patients at 12 months, 1.9% (95% CI 1.4–2.4%) at 24 months, and 7.9% (95% CI 6.4–9.4%) at 60 months (Fig. 10).

### Recurrence in marsupialisation

Data on recurrence and follow-up times pertaining to 343 patients treated for PSD by marsupialisation were extracted from 8 RCTs^17,18,30,47,96,101,603,604^. Among these patients, a recurrence of 1.0% (95% CI 0.0–2.3%) was observed in patients at 12 months and 14.3% (95% CI 0.0–30.3%) at 24 months.

Data on recurrence and follow-up times pertaining to 3,207 patients treated for PSD by other flap techniques were extracted from 55 additional non-RCTs^4,108,109,115,129,132,133,137,143,163,170,171,177,188,190,193,200,204,215,218,224,230,245,272,279,294,303,313,315,317,318,336,352,358,363,369,593,605–622^. Among these patients, a recurrence of 1.8% (95% CI 1.2–2.3%) was observed in patients at 12 months, 5.6% (95% CI 4.5–6.7%) at 24 months, 9.4% (95% CI 7.6–11.1%) at 60 months, and 16.3% (95% CI 11.8–20.9%) at 120 months (Supplementary Fig. 1).

### Recurrence in limited excision

Data on recurrence and follow-up times pertaining to 384 patients treated for PSD by limited excision were extracted from 5 RCTs^29,50,105,604,623^. Among these patients, a recurrence of 1.3% (95% CI 0.0–2.9%) was observed in patients at 12 months and 1.7% (95% CI 0.0–3.5%) at 24 months.

Data on recurrence and follow-up times pertaining to 6,366 patients treated for PSD by limited excision PSD treatment were extracted from 71 additional non-RCTs^52,61,69,72,73,75,81,83,88,94,106,114,119,121,124,125,131,140,142,144,149,160,208,221,226,248,249,261,285,293,295,319,321,322,400,410,416,536,609,625–656^. Among these patients, a recurrence of 5.0% (95% CI 4.3–5.6%) was observed in patients at 12 months, 6.8% (95% CI 6.0–7.7%) at 24 months, 16.2% (95% CI 14.3–18.2%) at 60 months, and 34.0% (95% CI 26.3–41.6%) at 120 months (Supplementary Fig. 2).

### Recurrence in pit picking

Data on recurrence and follow-up times pertaining to 98 patients treated for PSD by pit picking were extracted from 2 RCTs^85,86^. Among these patients, a recurrence of 4.3% (95% CI 0.0–8.7%) was observed in patients at 12 months and 8.3% (95% CI 0.0–17.0%) at 24 months.

Data on recurrence and follow-up times pertaining to 6,272 patients treated for PSD by pit picking were extracted from 32 additional non-RCTs^179,329,354,410,412,483,655–680^. Among these patients, a recurrence of 2.7% (95% CI 2.2–3.1%) was observed in patients at 12 months, 6.5% (95% CI 5.7–7.3%) at 24 months, and 15.6% (95% CI 13.8–17.4%) at 60 months (Supplementary Fig. 3).

### Recurrence in partial closure

Data on recurrence and follow-up times pertaining to 73 patients treated for PSD by partial closure were extracted from 1 RCT^48^. Due to the single observation, meta-analysis based on RCTs was not possible.

Data on recurrence and follow-up times pertaining to 530 patients treated for PSD by partial closure were extracted from 11 additional non-RCTs^118,124,125,155,167,168,182,315,681–683^. Among these patients, a recurrence of 2.8% (95% CI 1.2–4.4%) was observed in patients at 12 months, 5.1% (95% CI 2.8–7.3%) at 24 months, and 19.0% (95% CI 12.7–25.4%) at 60 months (Supplementary Fig. 4).

### Recurrence in incision and drainage

No RCTs are available that report recurrence and follow-up times for incision and drainage.

Data on recurrence and follow-up times pertaining to 360 patients treated for PSD by incision and drainage were extracted from 13 non-RCTs^118,162,177,179,197,199,206,254,264,378,684–686^. Among these patients, a recurrence of 10.4% (95% CI 6.6–14.3%) was observed in patients at 12 months, 25.9% (95% CI 19.1–32.8%) at 24 months, and 40.2% (95% CI 29.4–50.9%) at 60 months (Supplementary Fig. 5).

### Recurrence in phenol treatment alone

Data on recurrence and follow-up times pertaining to 70 patients treated for PSD by phenol alone were extracted from 1 RCT^12^. Due to the single observation, meta-analysis was not possible.

Data on recurrence and follow-up times pertaining to 1,947 patients treated for PSD by phenol alone were extracted from 26 additional non-RCTs^194,219,338,355,445,457,687–706^. Among these patients, a recurrence of 1.9% (95% CI 1.1–2.6%) was observed in patients at 12 months, 14.1% (95% CI 11.8–16.5%) at 24 months, and 40.4% (95% CI 27.8–52.9%) at 60 months (Supplementary Fig. 6).

### Recurrence in laser alone

No RCTs are available that report recurrence and follow-up times for PSD treatment by laser alone.

Data on recurrence and follow-up times pertaining to 125 patients treated for PSD by incision and drainage were extracted 14 non-RCTs^175,707–719^. Among these patients, a recurrence of 1.9% (95% CI 0.0–4.7%) was observed in patients at 12 months, 5.1% (95% CI 0.4–9.8%) at 24 months, and 36.6% (95% CI 3.8–69.4%) at 60 months (Supplementary Fig. 7).

Comparing recurrence at 60 month following surgery amongst the RCT studies (11,730 patients, Figure 2, column “60”), range was 10.2%–21.9%; separated by a factor of 2.1. In all studies (Figure 3), range was from 1.9% to 40.4% at 60 months, giving a factor of 40.4/1.9 = 21. Thus, recurrence results differ by a factor of 2.1 in RCTs and by a factor of 21 in all studies through selection of a surgical therapy (when a given 5 year follow up is applied).

On the other hand, recurrence varies with time since surgery. In RCT primary open results (as shown in Figure 2), line primary open treatment, recurrence is 1.0% at 12 months and increases to 16.5% at 60 months. Recurrence varies by a factor of 16,5/1 through time in this primary open group, while this is lower in the Karydakis & Bascom group (10,2/1,5; 6,8).

In the 89,583 patients group combining RCT and non-RCT data, the quotient between longest and shortest recurrence result can be found in the Limberg & Dufourmentel group, with 0.4% at 12 months and 11.4% at 120 months following surgery (factor 28,5). Thus, recurrence results may differ by a factor of 6, 8 and more for a given surgical therapy if follow up time is not properly defined (or defined at all). This indicates that a thorough evaluation of a procedure in view of recurrence has to include the specific relation of recurrence to follow-up time.

## Discussion

We have reviewed all pilonidal sinus treatment data from all available cases from the first published description of pilonidal sinus in 1833 to the present day. We have analysed data from more than 80,000 patients surgically treated for a single disease over the past 180 years, and have systematically assessed the relationship between recurrence, follow-up times, and surgical procedure. Our meta-analysis of the data identified a relationship between recurrence of PSD and follow-up times for each different therapeutic approach. The mean recurrence for all cases measured at the one-year time point differed from the five-year recurrence by a factor of 5; whereas, for the individual therapeutic procedures, one and five-year recurrence differed by a factor of 3 to 20 (Tables 2 and 3).

Recurrence varies between surgical procedures by a factor of 2,1 at 60 months in RCTs and 21 in all studies. On the other hand, recurrence varies by a factor of 19 to 29 within a given surgical therapy if comparing 12 months and longest follow up results (Tables 2 and 3). Thus, any report of a given surgical procedure without specified follow up may contain a10 fold higher or lower true recurrence as the postulated one.

Our study has several limitations that we identify as follows: The systematic review and meta-analysis was limited to reports in English, French, German, Italian, and Spanish. Publications in other languages were included where definitive treatment and recurrence at specific follow-up times were described in an English abstract. Consequently, Japanese and Chinese literature is not fully included. On the other hand, pilonidal sinus is exceptionally rare in the Asian population^745^, therefore, we do not anticipate an emergence of Asian data that would significantly skew the current results. Further, our strategy of including all available evidence since January 1^st^, 1833 ensured a broad picture. Although additional older literature may be found and studied, such literature is unlikely to add significantly to the findings reported here, as regular follow-up was not commonplace in early times; surgeons relied more on “return on recurrence” follow-up^746,749^. Furthermore, the older literature includes more primary open treatment methods with follow-up exceeding 120 months^166,750^, which are well-represented in the analysis. Therefore, the inclusion of additional new primary open patients is unlikely to change our findings. Given the design, the scope of this study is relatively narrow and specific. Yet, including more variables such as e.g. length of hospital stay, length of post-op course, and cost of post-op course would be a very extensive beyond the scope of a single study. Another source of bias may be our strategy for grouping therapeutic procedures. We had to condense multiple, sometimes minute, variants of mainstream surgical therapies into cohorts large enough for thorough analysis. While some therapies are gone, and will not return, others, such as marsupialisation, seem to be enjoying a resurgence, and new strategies such as endoscopic approaches are currently being investigated. Additionally, studies were picked regardless of their inclusion of primary or recurrent PSD cases what may affect the surgical technique of choice and the outcome in many ways. Statistical limitations are built into the design of this study. While detailed information about a single patient is commonly not available, it was necessary to simulate single patients from pools of patients reported in each single study. Therefore, no sub-analysis of links between recurrence and follow-up time for gender or age groups, etc., could be applied. Since the analysed studies reported follow-up times with various statistical measures (mean, median, and range), there could be a potential bias within the details in the survival curve structures. Abrupt drops, for example, are often due to studies in which large numbers of patients experienced recurrence at a specific (mean/median/centre of range) follow-up time. Therefore, the specific drops in the survival curves should be interpreted cautiously. For the purpose of this study, the goal was to acquire an understanding of general links between follow-up times and recurrence among therapeutic procedures. Although we cannot necessarily determine, by extrapolation from our results, a particular follow-up time at which recurrence increases, the trend over time is meaningful, and thanks to the very high case numbers, is reliably estimated.

The highest incidence of recurrence identified in our data, 67.9%, occurred 240 months after primary midline closures; so, this method should be abandoned straight away, while other traditional approaches such as primary open treatment can be justified, also when considering the development of more complicated surgeries involving flap techniques. Correctly, this leads to a call for more off-midline closure education for surgeons^751^. From our analysis of data from more than 80,000 patients over 18 decades, we found that the Karydakis and the Bascom cleft lift procedures show the lowest recurrence at any time of follow-up, followed by rhomboid flaps and other flaps. However, current RCT evidence only provides follow-up data for 10 years postoperatively for Karydakis/Bascom, and a longer-term study would be of interest. Nevertheless, non-RCT data point into the direction of a continued low recurrence with these advancement and rotational flap methods. Because of the high recurrence as early as 2 years (25.9%, rising to 40.2 after 5 years), incision and drainage cannot be recommended as definite therapy. The simple manoeuvre easily relieves a patient from pain in an acute situation, but must be followed by a definite technique with lower recurrence. Similarly, phenol treatment is associated with high recurrence, 14.1% at 24 months and 40.4% at 60 months; longer term follow-up data are unavailable. A Turkish group has reported on the phenol technique recently^457^. They praise its minimal invasiveness and concomitant short duration of stay in hospital. However, given the high recurrence, these might be misleading arguments. The data on laser treatment still remain weak; long term follow-up data and extensive cohorts have not yet been published.

Our results presented here suggest that the variances in recurrence are understandable once the follow-up time is taken into account. We found that recurrence is a function of follow-up time for every major surgical and non-surgical method analysed. Our endeavours here have established a respective benchmark, potentially increasing the comparability as also proposed, e.g., for a staging system^410^.

In conclusion, physicians must keep in mind that recurrence of surgical procedures in PSD impressively depend on follow-up time. This dependence, i.e. the steepness of increase of recurrence with longer follow-up times, is specific to a surgical procedure. Applying a recurrence without knowing the time since surgery may be open to a bias factor of up to 18 and above. The choice of surgical therapy influences recurrence by a factor of up to 21. As we are now able to understand recurrence in PSD in a more standardised way. Primary midline closure is dead, while older therapies (such as marsupialisation) may be reconsidered, while advancement flap (Karydakis & Bascom) and rotational flap procedures (Limberg/Dufourmentel) are undoubtedly primary league with asymmetrical procedures, as proven by RCT and combined RCT/non-RCT analysis in 89,583 patients available from 1833 to 2017. Follow-up of PSD patients should always be planned long term, i.e., five or ten years if reliable conclusions are to be drawn regarding the efficacy of a new procedure or on the efficacy of your own results using an already known technique.

## Methods

### Ethical approval and informed consent

The systematic review with meta-analysis and merged data analysis included no experiment carried out on live vertebrates (or higher invertebrates), humans or human samples. Thus, formal ethics approval was not required.

### Search strategy and study selection criteria

To assemble a comprehensive database pertaining to PSD, we systematically searched for the NCBI Medical Subject Heading (MeSH) term, “pilonid*“, as well as “dermoid” AND “cyst” in MEDLINE, PubMed, PubMed Central, Scopus, Ovid, Embase, and Cochrane Central Register of Controlled Trials (CENTRAL). We additionally searched these terms in Google, Google Scholar, ResearchGate, and references listed in national and international guidelines such as the S3 guidelines of the Association of the Scientific Medical Societies in Germany on the treatment of PSD. We also assessed the references listed in the literature cited of all documents retrieved by the searches. Documents retrieved included randomised, non-randomised, prospective, retrospective, and observational studies such as cohort, case-control, cross-sectional studies, and case reports published between 1833 to 2017. Four authors (VS, MML, MD, and DD) reviewed the retrieved documents for compliance with the inclusion criteria: specification of the definitive treatment, recurrence, and length of follow-up. Reports in English, French, German, Italian, and Spanish were considered, as were publications in other languages if definitive treatment and recurrence at specific follow-up times were described in an English abstract, or if the authors contacted via email or Research Gate provided an English translation of their surgical approach, recurrence, and follow-up time. Exclusion criteria were: PSD in other than presacral location, neoplasia involvement, double publication of data by an author. Further, studies lacking any component of the minimal data set which comprised of: definitive treatment strategy/recurrence/follow-up time were excluded. Prior meta-analysis reports and review articles were excluded, as well, although their reference lists were screened for potential additions to the evidence. Also, previously unpublished data presented in review articles were taken into account. Studies in which recurrence were deduced from patients returning with recurrent disease (“return on recurrence”), but which did not actively investigate the majority of non-returners, were excluded.

The review protocol is registered in the National Health Service (NHS) international prospective register of systematic reviews PROSPERO (42016051588).

### Data collection, extraction and quality assessment

All studies were analysed and documented on paper. The transcript data were collected into a Microsoft Excel (Version 2016, Microsoft Corp., Redmond, WA) spreadsheet, and correct transfer was controlled. Every specific therapeutic strategy reported in a paper was assigned to a line. Columns included citation details, number of patients included, therapeutic procedures, reported follow-up times, study details and recurrence.

The statistical measures applied for reporting of follow-up times are not standardised, and thus, were not identical among different studies. However, given the relative clustering of the disease incidence in young adults, mean and median reports were treated as equivalent. For data that included a range of follow-up times, the centre of the given range was used in our analysis. For data in which minimum follow-up times were reported, the values were integrated as is.

Individual studies were assessed for consistency in described methods and reported results to minimize potential risk of bias in a thorough data synthesis. A subgroup of prospective randomized control trials was analysed separately in addition to check for consistency with the complete set of studies. The reported recurrence in each study were then linked to the study’s follow-up time. Follow-up time was defined as mean, median, centre of range, or minimum. To compare information across all studies, single patients were statistically simulated. I.e., for each study participant, a data sample was extracted containing recurrence, follow-up time, and therapeutic procedure. If, for example, a study included 500 patients and a recurrence of 20% for a particular therapeutic procedure, then 100 single samples would be defined as recurrent disease, whereas, the remaining 400 samples would be defined as recurrence-free. In this simulation, some information, such as gender ratios, could not be included since this information was only available cumulatively in the majority of studies.

In cases where an article addressed more than one therapeutic strategy, the data pertaining to each treatment strategy were considered separately in our analysis.

### Grouping of therapeutic procedures and statistical analyses

Therapeutic procedures were analysed cumulatively (“overall”) and stratified as subgroups (Table 1). The overall analysis also included some other different techniques not specifically analysed as subgroup. For statistical analysis and visualizations, the statistical software package R (version 3.1.0) in the R-studio framework (version 0.98.982) was used. Statistical significance was assumed if p < 0.05. All tests were considered in a two-tailed set-up. For the analysis of recurrence free outcome over time, survival analysis according to Kaplan-Meier including pointwise 95% confidence intervals (CI) was used as implemented in the R-package ‘survival’ (version 2.40-1). These analyses were performed for each defined therapeutic procedure. Results were plotted as percent of recurrence-free outcomes with their 95% CI. In order to comprehend the data leading to the Kaplan-Meier curves, the numbers of patients included in the intervals of 0-12, 12-24, 24-60, 60-120, and > 120 months are included on the horizontal axes of the plots. If no specific data were available for an interval, linear interpolation of recurrence free outcome according to the two nearest observed follow-up times was used. To provide a comprehensive study on recurrence and a rational basis for selecting treatment procedures, we assessed data in the manner of a classical meta-analysis of RCTs, and further employed an analysis that included non-RCTs in the manner of a merged data analysis. Because the Kaplan-Meier curves were calculated as stepwise functions, small discrepancies between the plotted and the tabled values may occur.

Potential heterogeneity of the study outcomes was assessed through Cochrane analysis and I^2^ calculation as described previously^752,753^. Therefore, the articles were grouped according to procedures and follow-up time-intervals as applied for plotting the Kaplan-Meier curves. A separate analysis was conducted for studies based on randomized controlled trials (RCTs) and for the complete set of studies considered (RCTs and non-RCTs). The data used for computing Cochrane’s Qs were computed with the recurrence as reported in the corresponding studies, weighted with the respective numbers of study participants. For assessing significance of heterogeneity, a Chi^2^ test was employed. I^2^ was computed to complete the results of the Chi^2^ test.

### Data Availability Statement

All data and calculations are available to readers upon request to the corresponding author.

### Ethics

This article does not contain any studies with human participants, human samples or live vertebrates. Therefore, no informed consent had to be obtained prior to preparation of current manuscript.

## Electronic supplementary material

Supplemental Figures 1–7
