# Supplementary material for: Common surgical procedures in pilonidal sinus disease: A meta-analysis, merged data analysis, and comprehensive study on recurrence
Source: Sci Rep. 2018 Feb 15;8:3058. doi: 10.1038/s41598-018-20143-4 (PMC5814421; doi:10.1038/s41598-018-20143-4)
Supplement: Supplementary file 1 — Supplemental Figures 1–7 [file 41598_2018_20143_MOESM1_ESM.pdf]

Stauffer VK, Luedi MM, Kauf P, Schmid M, Diekmann M, Wieferich K, Schnüriger B, DollD: *Common surgical procedures in pilonidal sinus disease: A meta-analysis, merged data analysis, and comprehensive study on recurrence*

Supplemental Figures

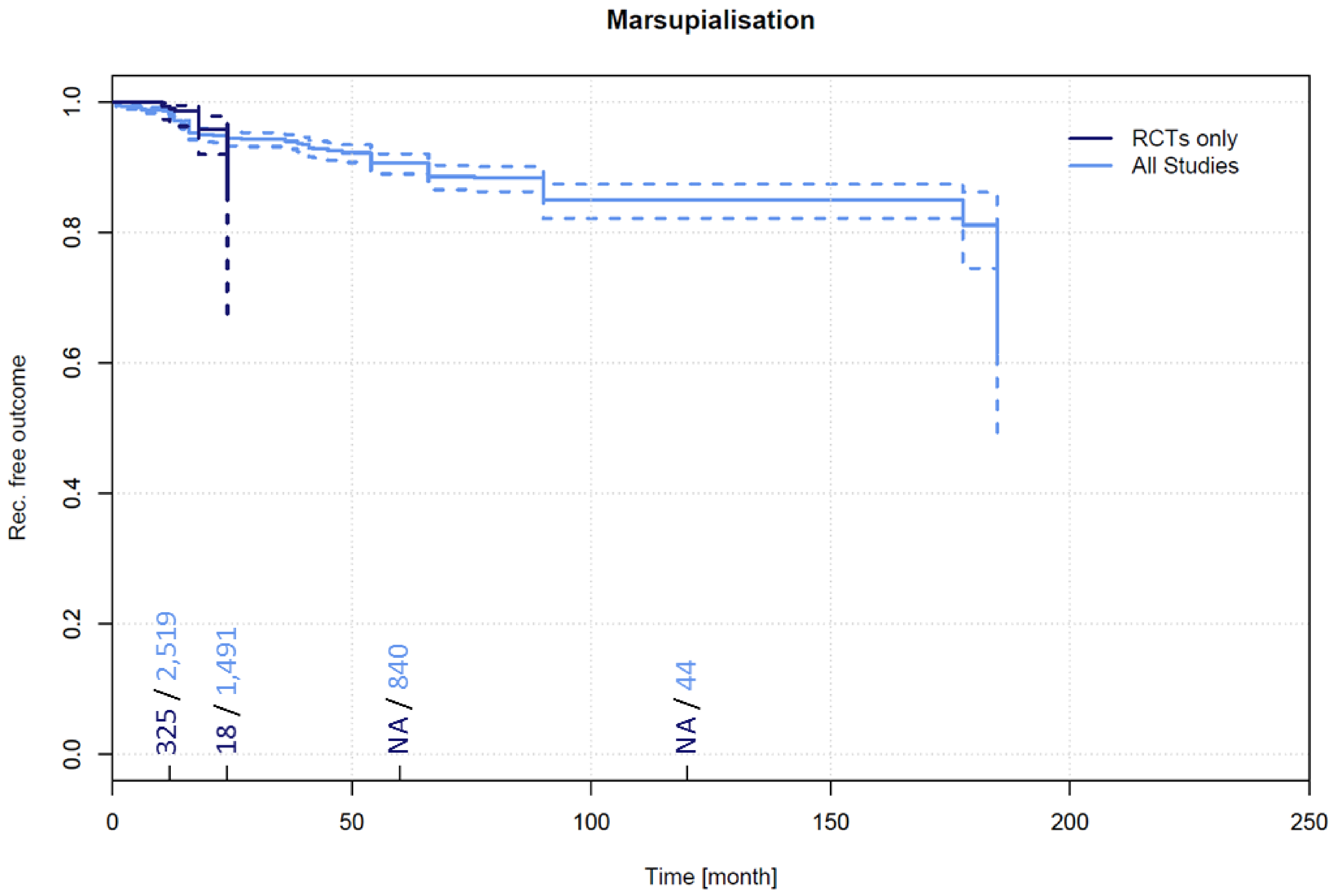

Supplemental Figure 1: Recurrence free outcome as a function of follow-up time of patients treated by marsupialisation. Data presented are for RCTs only and for all available studies. Numbers of patients included in the analysis are indicated at 12, 24, 60, and 120 months. Dashed lines indicate 95% confidence intervals

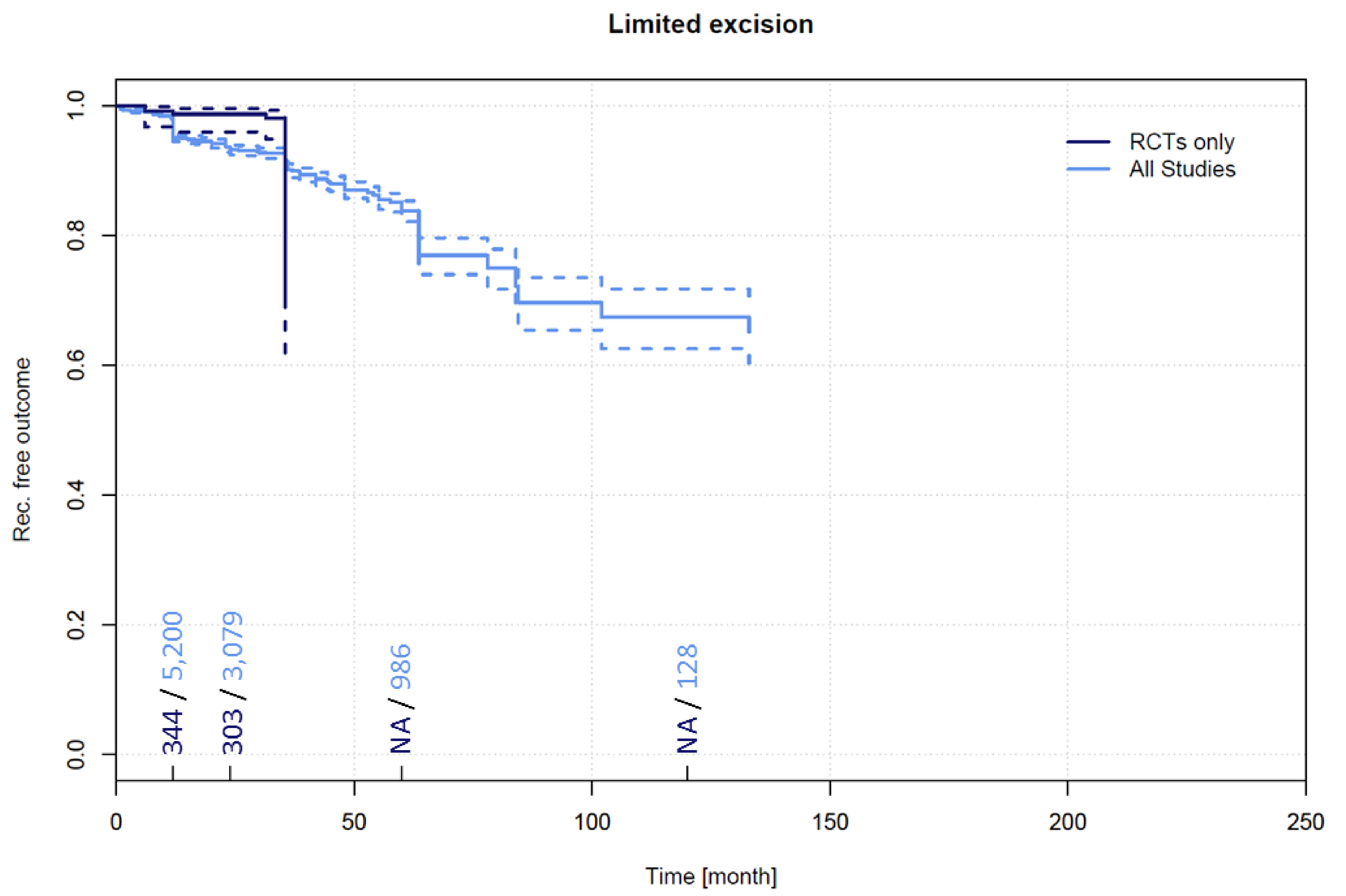

Supplemental Figure 2: Recurrence free outcome as a function of follow-up time of patients treated with limited excision. Data presented are for RCTs only and for all available studies. Numbers of patients included in the analysis are indicated at 12, 24, 60, and 120 months. Dashed lines indicate 95% confidence intervals.

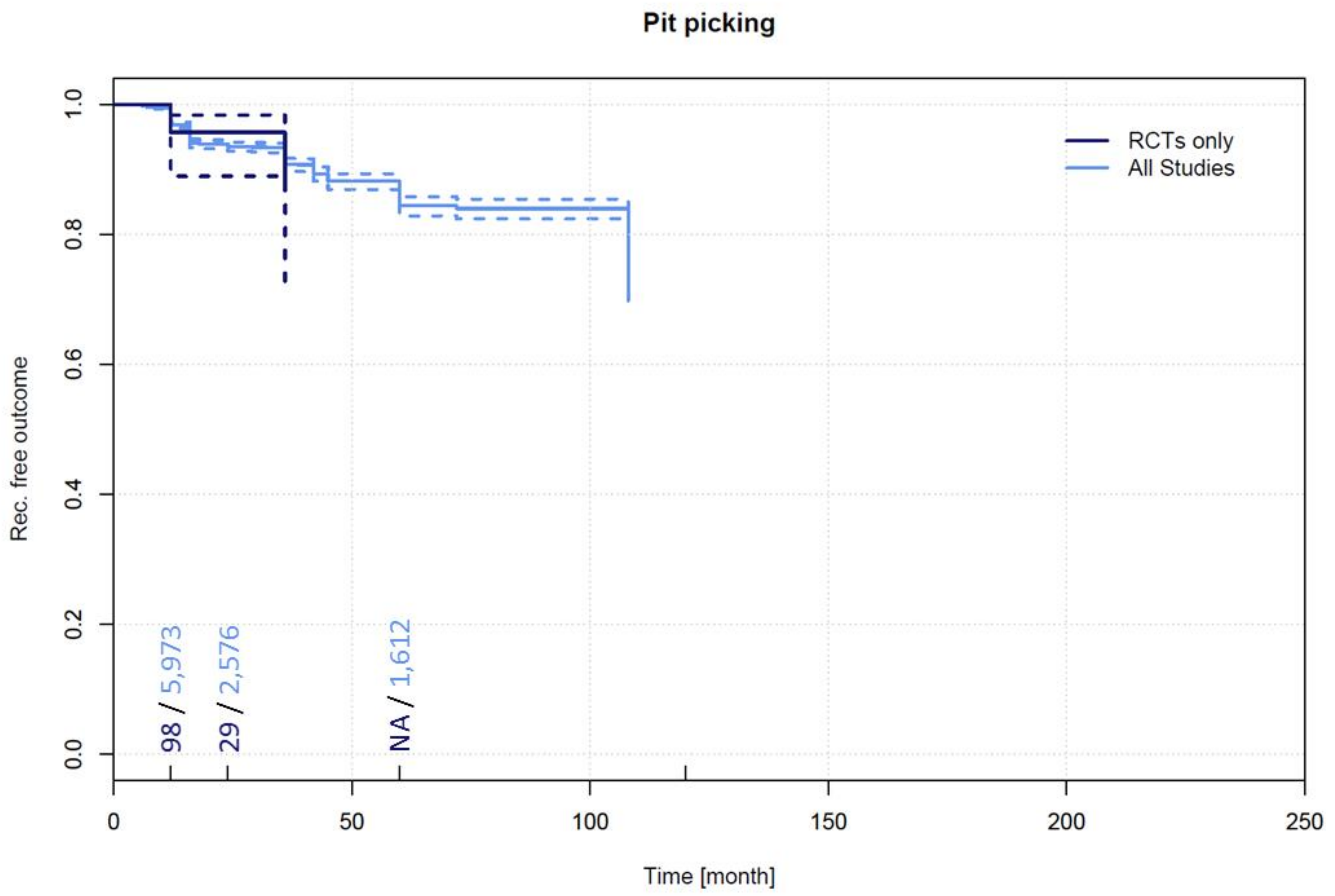

Supplemental Figure 3: Recurrence free outcome as a function of follow-up time of patients treated with pit picking. Data presented are for RCTs only and for all available studies. Numbers of patients included in the analysis are indicated at 12, 24, and 60 months. Dashed lines indicate 95% confidence intervals.

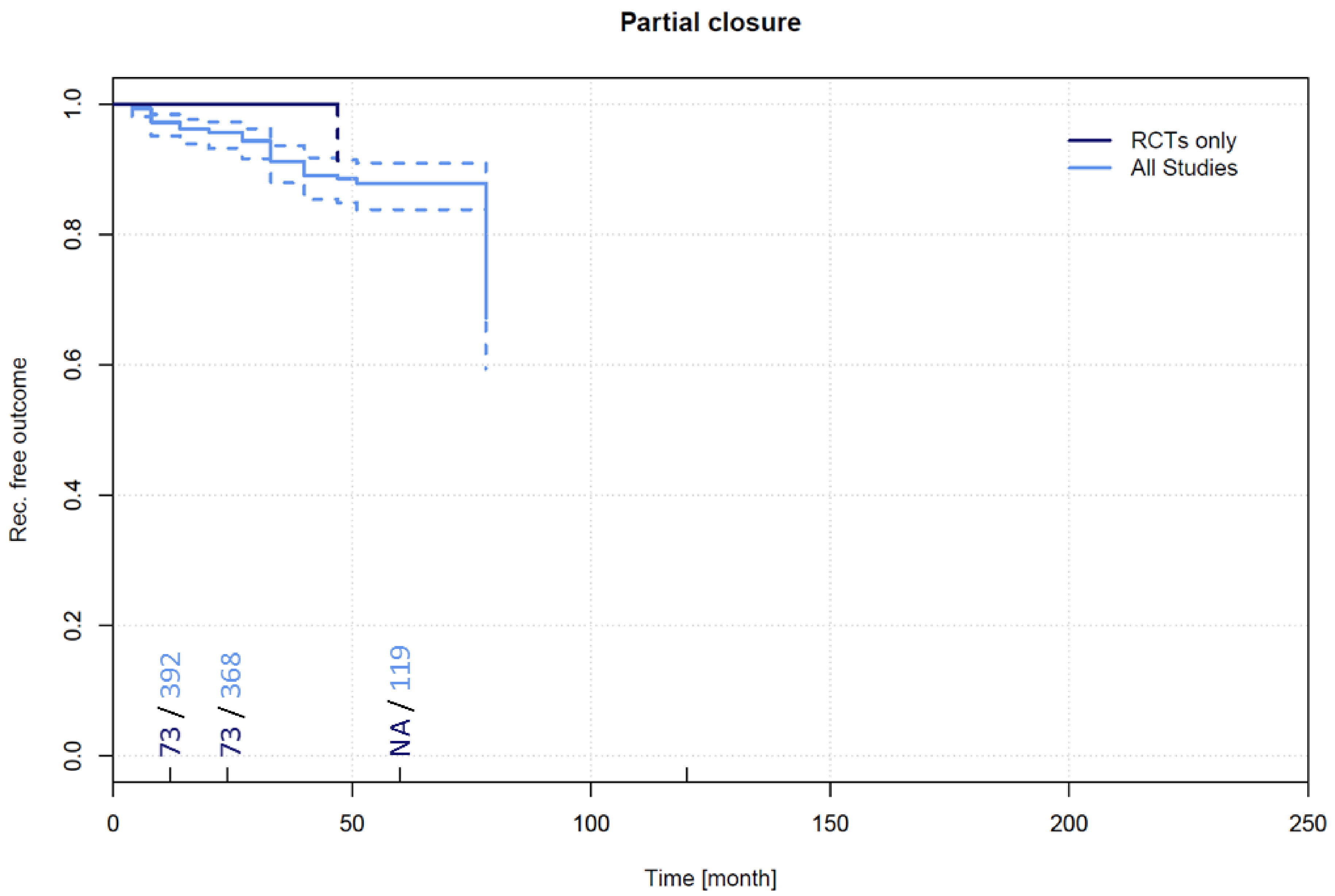

Supplemental Figure 4: Recurrence free outcome as a function of follow-up time of patients treated with partial closure. Data presented are for RCTs only and for all available studies. Numbers of patients included in the analysis are indicated at 12, 24, 60, and 120 months. Dashed lines indicate 95% confidence intervals.

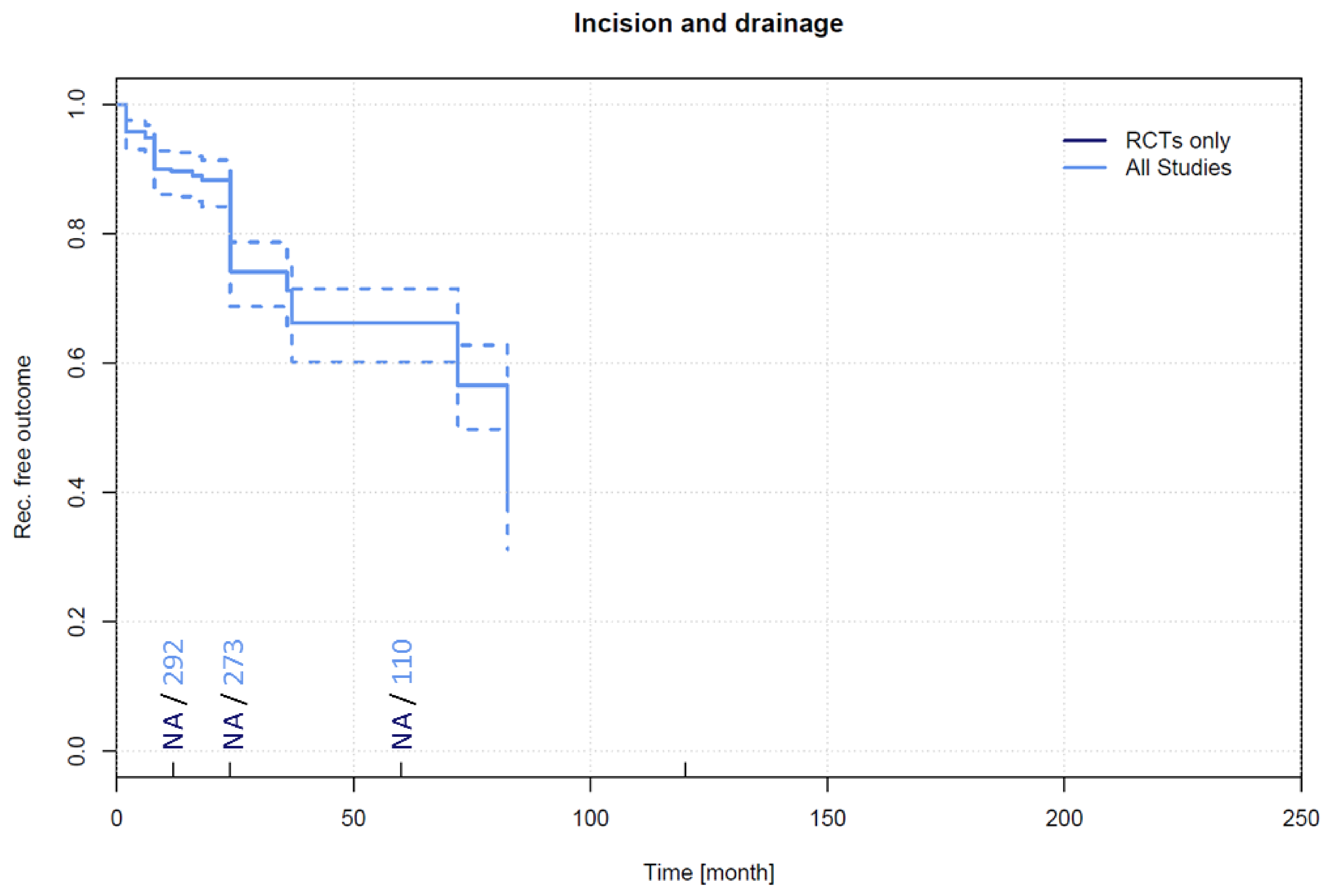

Supplemental Figure 5: Recurrence free outcome as a function of follow-up time of patients treated with incision and drainage. Data presented are for RCTs only and for all available studies. Numbers of patients included in the analysis are indicated at 12, 24, 60, and 120 months. Dashed lines indicate 95% confidence intervals.

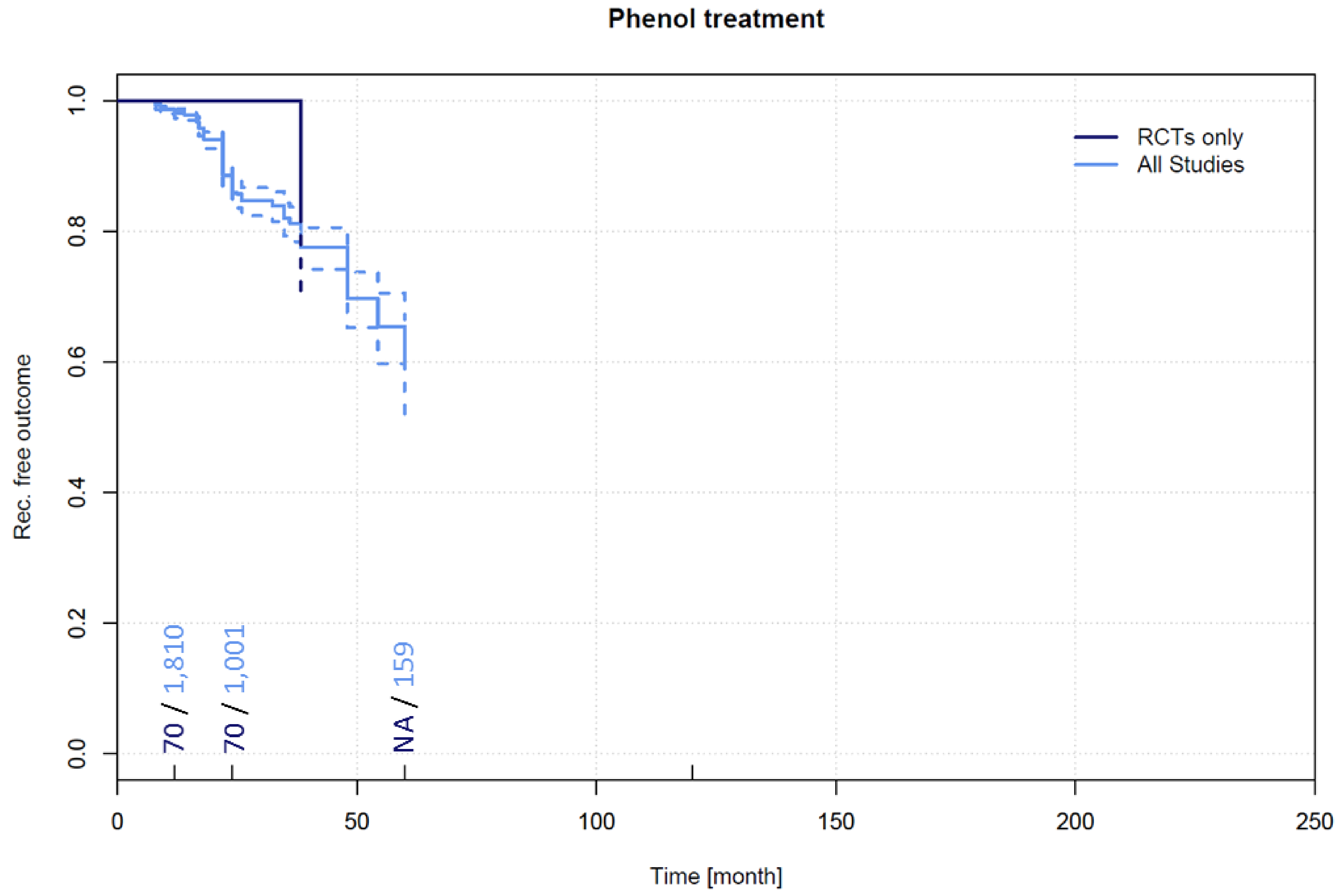

Supplemental Figure 6: Recurrence free outcome as a function of follow-up time of patients treated with phenol treatment alone. Data presented are for RCTs only and for all available studies. Numbers of patients included in the analysis are indicated at 12, 24, and 60 months. Dashed lines indicate 95% confidence intervals.

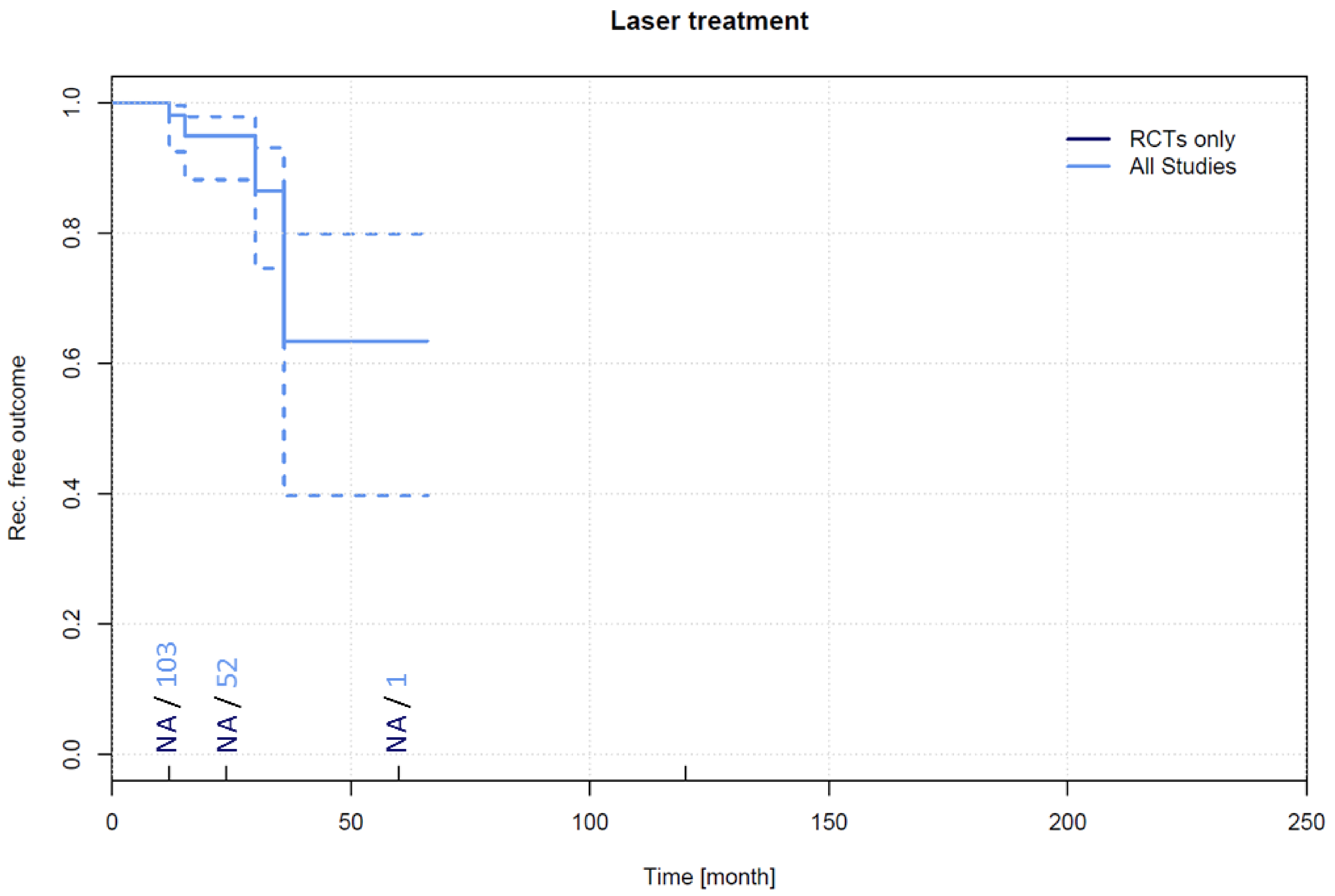

Supplemental Figure 7: Recurrence free outcome as a function of follow-up time of patients receiving laser treatment alone. Data presented are for RCTs only and for all available studies. Numbers of patients included in the analysis are indicated at 12, 24, and 60. Dashed lines indicate 95% confidence intervals.
